# Exploring doctors’ perspectives on generative-AI and diagnostic-decision-support systems

**DOI:** 10.1136/bmjhci-2024-101371

**Published:** 2025-07-23

**Authors:** Saba Esnaashari, Youmna Hashem, John Francis, Deborah Morgan, Anton Poletaev, Jonathan Bright

**Affiliations:** 1 The Alan Turing Institute, London, UK; 2 University of Bath, Bath, UK; 3 Zug Institute for Blockchain Research (ZIBR), University of Lucerne, Lucerne, Switzerland

**Keywords:** Artificial intelligence, BMJ Health Informatics

## Abstract

This research presents key findings from a project exploring UK doctors’ perspectives on artificial intelligence (AI) in their work. Despite a growing interest in the use of AI in medicine, studies have yet to explore a representative sample of doctors’ perspectives on, and experiences with, making use of different types of AI. Our research seeks to fill this gap by presenting findings from a survey exploring doctors’ perceptions and experiences of using a variety of AI systems in their work. A sample of 929 doctors on the UK medical register participated in a survey between December 2023 and January 2024 which asked a range of questions about their understanding and use of AI systems.

Overall, 29% of respondents reported using some form of AI in their practice within the last 12 months, with diagnostic-decision-support (16%) and generative-AI (16%) being the most prevalently used AI systems.

We found that the majority of generative-AI users (62%) reported that these systems increase their productivity, and most diagnostic- decision-support users (62%) reported that the systems improve their clinical decision-making. More than half of doctors (52%) were optimistic about the integration of AI in healthcare, rising to 63% for AI users. Only 15% stated that advances in AI make them worried about their job security, with no significant difference between AI and non-AI users. However, there were relatively low reported levels of training, as well as understandings of risks and professional responsibilities, especially among generative-AI users. Just 12% of respondents agreed they have received sufficient training to understand their professional responsibilities when using AI, with this number decreasing to 8% for generative-AI users. We hope this work adds to the evidence base for policy-makers looking to support the integration of AI in healthcare.

## Introduction

 Recent advances in artificial intelligence (AI) have put increasing focus on the use of AI in the workplace. In the field of medicine, reports have highlighted AI’s potential use in areas such as diagnostics, system efficiency and preventative medicine.^1^ Indeed, many AI tools are already available to medical practitioners. Specialised diagnostic-decision-support systems such as RapidAI and Brainomix are used by doctors to aid in the analysis of brain scans and help triage stroke patients; AI-driven logistic systems such as Navenio’s Intelligent Workforce Solution (IWS) promote system efficiency by managing medical staff’s schedules and tasks; and recently multipurpose generative AI tools such as ChatGPT provide a new way to help doctors across a wide range of tasks, such as to draft case reports, reword medical diagnoses to simplify communications for patients, as well as aid in the preparation of presentations.

In the UK, a survey found that one in five general practitioners reported using generative AI in clinical practice^2^ while another survey of doctors in training found that the majority experienced positive impacts from AI technologies on their training and education.^3^ Similarly, a qualitative study conducted with both clinical and non-clinical National Health Service^1^ staff showed prevalent enthusiasm around AI’s potential to better facilitate patients’ access to care.^4^ Despite this, studies have yet to explore a representative sample of doctors’ perspectives on, and experiences with, making use of different types of AI. Our research seeks to fill this gap by presenting findings from a survey exploring doctors’ perceptions and experiences of using a variety of AI systems in their work.

## Methods

A random sample of approximately 30 000 doctors was drawn from all registered doctors on the UK’s medical register and contacted for the survey. 929 doctors consented and completed the survey from December 2023 to January 2024. In the survey, which took approximately 15 min to complete, respondents were asked a range of questions about their perceptions of, and experiences with, AI systems. An important element of this work was going beyond researching AI as a generic tool, and instead looking at different types of AI systems. Hence, we distinguished between three key types of AI systems and provided respondents with a definition of each type:

Diagnostic-decision-support: Systems that directly support medical decision-making by, for example, identifying medical conditions or predicting risk.

System efficiency: Systems that try to optimise the internal workings of a healthcare service by, for example, predicting missed appointments or forecasting staff levels.

Generative AI: Systems such as ChatGPT that create text or images, that could be used, for example, to draft responses to patient queries.

To improve its representativeness, the sample was weighted by respondents’ age, gender, where they obtained their primary medical qualification: UK, EU or other countries, and their registration status. For more information on demographic characteristics of respondents and the survey, please refer to online supplemental file 1 and online supplemental file 2. The results are supported by significant coefficients in regression analysis of responses on demographic characteristics, AI use and AI type (online supplemental file 3. In this paper, we present the differences observed between perspectives of AI users versus non-AI users, and generative-AI versus diagnostic-decision-support users.^1^

## Results

Overall, 29% of respondents reported using some form of AI system in their practice within the last 12 months (n=270). Diagnostic-decision-support (16%, n=145) and generative AI (16%, n=143) were most commonly used, with lower reported usage of system-efficiency or other types of systems (7%, n=65). Men and younger doctors were more likely to report using generative AI compared with women and older doctors, with no significant differences between usage of diagnostic-decision-support for these groups. Across specialties, doctors working in radiology were more likely to report using AI (48%) while those in psychiatry were the least likely to have reported using any AI system (11%). For more information on AI usage, refer to online supplemental file 1.

More than 60% of doctors using AI were optimistic about the integration of AI in healthcare (63%) while this number was just below half for non-AI users (47%). Male doctors reported more optimism compared with females (61% vs 43%).

Just 15% of all doctors stated that advances in AI make them worried about their job security, with no significant difference between AI and non-AI users. Just 12% of all respondents agreed they have received sufficient training to understand their professional responsibilities when using AI (table 1), with this figure decreasing to 10% for non-AI users, 8% for generative-AI users and 8% for those qualified in the UK (online supplemental file 4).

**Table 1:** Aggregate responses to perception(table 1) and responsibility questions for all doctors, AI users and non-AI users

Group	Question (agree/neutral/disagree)	Choice	All: % of n=929	AI users: % of n=270	Non-AI users % of n=659
Perception	I am optimistic about the integration of AI systems in healthcare	Optimistic	52	63	47
		Neutral	24	19	26
		Pessimistic	24	18	27
	Advances in AI are making me worried about my job security	Agree	15	11	16
		Neutral	19	20	18
		Disagree	67	69	65
Responsibility	I've had sufficient training to understand my professional responsibilities when using AI	Agree	12	17	10
		Neutral	18	26	15
		Disagree	70	57	75
	I understand the risks of AI in healthcare in my area of practice	Agree	44	53	40
		Neutral	24	24	25
		Disagree	32	23	35
	I understand who is responsible if a decision is made incorrectly involving an AI system	Agree	30	37	28
		Neutral	19	22	18
		Disagree	51	42	54

The choices were: strongly agree, agree, neutral, disagree, strongly disagree, NA. We integrated agree and strongly agree, disagree and strongly disagree, and NA and neutral in this table. Refer to online supplemental file 2 and online supplemental file 3 for more details.

AI, artificial intelligence.

AI users were asked a series of questions about how they interact with their chosen (most frequently used) system. We highlight the difference between perspectives of those who chose generative AI versus those who chose diagnostic-decision-support as their primary or most frequent AI system in table 2.

**Table 2:** Aggregate responses to experience questions for AI users

Question (agree/neutral/disagree)	Choice	AI users:% of n=270	diagnostic-decision-support system users: % of (n=126)	Generative-AI users: % of n=113
The system has increased my productivity	Agree	53	45	62
	Neutral	31	33	29
	Disagree	16	23	9
The system improves my clinical decision making	Agree	42	62	19
	Neutral	41	28	54
	Disagree	17	11	26
I have received sufficient training on the system	Agree	38	56	15
	Neutral	33	27	40
	Disagree	29	17	45
I understand how to raise any concerns I have about the system	Agree	35	44	20
	Neutral	29	25	36
	Disagree	35	31	44
If appropriate, I could explain the outputs of the system to patients	Agree	53	70	34
	Neutral	35	22	50
	Disagree	12	8	16

The choices were: strongly agree, agree, neutral, disagree, strongly disagree, NA. We integrated agree and strongly agree, disagree and strongly disagree, and NA and neutral in this table. Refer to online supplemental file 2 and online supplemental file 3 for more details.

AI, artificial intelligence.

The majority of generative-AI users (62%) agreed these systems increase their productivity, while less than half of diagnostic-decision-support users (45%) agreed with this. On the other hand, the majority of diagnostic-decision-support users (62%) reported that the systems improve their clinical decision-making, with this number being at just 19% for generative-AI users. More than half of diagnostic-decision-support users (56%) stated that they have received sufficient training on the system they are using compared with just 15% of generative-AI users. In addition, 70% of diagnostic-decision-support users stated they can explain the output of the systems to patients, compared with just 34% for generative-AI users. Nearly half of diagnostic-decision-support users (44%) stated that they understand how to raise concerns regarding AI systems, with this number being just 20% among generative-AI users (table 2 and online supplemental file 4).

## Discussion

Our findings illustrate that AI is embedded in the working life of over a quarter of doctors in the UK, with higher usage of generative-AI among male doctors than females, and no difference for diagnostic-decision-support usage across gender. This gender propensity in adoption suggests that there is room for further research which explores this discrepancy—the potential causes of which could be numerous. The equal use of diagnostic-decision-support between genders observed in our findings could, for example, be an indication that female doctors prefer to use established systems over emergent technologies like generative AI.

We find that there is general optimism for integration of AI in healthcare, as well as its potential for improving productivity and enhancing decision-making in clinical practice. This echoes research which found increased decision-making confidence among physicians with access to an AI-powered decision-making tool for stroke triage.^5^ Despite these positives, there are low reported levels of training, understanding of risks, professional responsibilities and explainability, especially among generative-AI users. This is important since we found that receiving training is associated with better reported understanding of how to raise concerns, as well as greater transparency between clinicians and their patients (online supplemental file 4); these findings point to the necessity of guidelines and training programmes for using AI in healthcare. Although recent developments in generative AI have paved new paths by enabling a bottom-up adoption approach,^6^ concerns around using these systems such as the transparency between patients and doctors and the uncertainty around the diagnosis/prognosis and how it should be raised require greater attention.

Our research has some limitations. For example, doctors who are more interested in AI may have been more willing to take the survey. Furthermore, although the data were collected after the emergence of generative-AI technologies like ChatGPT, it was before the full rollout of Copilot and other similar platforms, which are likely to have had an impact on use and perceptions of the technology.

However, generative AI might facilitate integration of diagnostic-decision-support, blurring the line between different types of AI systems defined here.^7^ We hope this work supports policy-makers looking to integrate AI in healthcare.

## Supplementary material

10.1136/bmjhci-2024-101371 online supplemental file 1

10.1136/bmjhci-2024-101371 online supplemental file 2

10.1136/bmjhci-2024-101371 online supplemental file 3

10.1136/bmjhci-2024-101371 online supplemental file 4

## Footnotes

The United Kingdom's National Health Service
