# Supplementary material for: Exploring doctors’ perspectives on generative-AI and diagnostic-decision-support systems
Source: BMJ Health Care Inform. 2025 Jul 23;32(1):e101371. doi: 10.1136/bmjhci-2024-101371 (PMC12306348; doi:10.1136/bmjhci-2024-101371)
Supplement: online supplemental file 1 [file bmjhci-32-1-s001.pdf]

Variable	Value	Frequency Sample (%) (n = 929)	Frequency GMC %	Frequency after imputation and Weighting % (n = 929)
**Gender (n_gmc = 315,771)**	**Female**	**38.75**	**49.5**	**49.5**
	Male	59.85	50.5	50.5
	Others	1.4	0	0
**Age (n_gmc = 315,771)**	Under 30	9.69	14.99	14.99
	30-39	29.5	34.68	34.68
	40-49	23.9	24.67	24.67
	50-59	22.71	16.99	16.99
	60 years and over	13.02	8.67	8.67
	NA	1.18	0	0
**Registration Status (n_gmc = 325,555)**	GP	18.52	21.27	21.27
	LED and SAS	23.68	28.8	28.8
	Specialist	33.58	27.8	27.81
	Trainee	20.56	22.13	22.13
	NA	3.66	0	0
**PMQ (n_gmc = 315,771)**	EEA	11.52	8.14	8.14
	IMG	32.51	33.69	33.69
	UK	55.97	58.17	58.17
**Speciality (n_gmc = 316,738)**	General Practice (No Speciality)	21.64	71.11	23.98
	Emergency Medicine	6.03	1.06	6.68
	Anaesthetics & Intensive Care	9.8	3.77	8.71
	Medicine	18.84	7.92	19.26
	Paediatrics	5.17	2.27	4.92
	Psychiatry	6.67	2.76	6.53
	Radiology	4.84	2.31	4.41
	Surgery	13.99	5	12.81
	Others	13.02	3.8	12.69

Table 1, Demographics of our sample versus GMC for Gender, age, registration status, PMQ, speciality with missing values imputed and weighting. Source: GMC website

Value	Variable	Any AI (%)	Generative AI (%)	DSS AI (%)
**All**	**All**	**29**	**16**	**16**
**Gender**	Female	26	12	16
	Male	33	19	16
**Age**	Under 40	31	17	16
	40-49	32	15	17
	50+	24	13	14
**Registration Status**	GP	28	16	15
	LED and SAS	29	16	13
	Specialist	36	18	21
	Trainee	24	12	15
**Specialty or area of practice**	Anaesthetics and Intensive Care Medicine	24	11	12
	Emergency Medicine	30	13	20
	General Practice	26	14	15
	Medicine[1]	37	20	19
	Paediatrics	27	19	11
	Psychiatry	11	8	0
	Radiology	48	11	40
	Surgery	34	20	16
**AI use Frequency (n = 270)**	Every day	26	8	38
	At least once a week	31	37	27
	At least once a month	16	18	15
	Less than once a month	15	25	7
	Don't know	13	13	13
**Common application areas of DDS systems (n = 126)**	Image processing		-	25
	Risk assessment and Triage		-	24
	Prognosis or Diagnosis		-	12
	Electrocardiogram (ECG) or Cardiotocography (CTG)		-	6
**Common types of generative AI used (n = 113)**	GPT		77	-
	Bard		8	-
	Accurx		4	
	Other (Claude, copilot, etc)		9	

[1] *This is an aggregate specialty group including general internal medicine, cardiology, rheumatology, and other specialties*

Table 2, AI use by demographics, AI system type, and frequency of use. Doctors could report using more than one system; Medicine is an aggregate specialty group including general internal medicine, cardiology, rheumatology, and other specialists.
