# Supplementary material for: Exploring doctors’ perspectives on generative-AI and diagnostic-decision-support systems
Source: BMJ Health Care Inform. 2025 Jul 23;32(1):e101371. doi: 10.1136/bmjhci-2024-101371 (PMC12306348; doi:10.1136/bmjhci-2024-101371)
Supplement: online supplemental file 2 [file bmjhci-32-1-s002.pdf]

Understanding doctors' perspectives on AI

Survey questions for the project "Understanding doctors' perspectives on AI", developed by The Alan Turing Institute. This material is made available under the Creative Commons Attribution 4.0 license. Please give appropriate credit when replicating this survey and indicate if changes were made. Please cite this survey as:

Understanding doctors' perspectives on AI. Survey. The Alan Turing Institute. 2024. Available at: https://github.com/ai-for-public-services/doctors-perspectives-ai

---

Questions on demographics

Which option best describes your current registration status?

If you are on the GP or Specialist register and currently on the training programme for a second speciality, please select 'Other' and write this in.

- ○ Licensed and in foundation year 1 training
- ○ Licensed and in foundation year 2 training
- ○ Licensed and in core training programme
- ○ Licensed and in GP training
- ○ Licensed and in specialty training
- ○ Licensed on the GP Register
- ○ Licensed on the Specialist Register
- ○ Licensed on both the GP and Specialist Register
- ○ Licensed and in specialist, associate specialist, or specialty (SAS) role
- ○ Licensed and in locally employed doctor role, e.g. clinical fellow, trust doctor, trust grade, etc.
- ○ Other (please write in) ____
- ○ Prefer not to say

What describes your main area of practice?
If you are currently in training, please answer this question based on your current role/post.

- ◯ Acute Medicine
- ◯ Anaesthetics
- ◯ Cardiology
- ◯ Clinical Oncology
- ◯ Dermatology
- ◯ Emergency Medicine
- ◯ Endocrinology and Diabetes Mellitus
- ◯ Gastroenterology and related disciplines
- ◯ General Internal Medicine
- ◯ General Practice
- ◯ General Surgery
- ◯ Geriatric Medicine
- ◯ Haematology/ haemato-oncology
- ◯ Intensive Care Medicine
- ◯ Medical Oncology
- ◯ Neurology
- ◯ Obstetrics and Gynaecology
- ◯ Occupational Medicine
- ◯ Ophthalmology
- ◯ Otolaryngology/ENT

- ◯ Paediatrics
- ◯ Pathology
- ◯ Plastic Surgery
- ◯ Psychiatry
- ◯ Public Health
- ◯ Radiology
- ◯ Renal Medicine
- ◯ Respiratory Medicine
- ◯ Rheumatology
- ◯ Trauma and Orthopaedic Surgery
- ◯ Urology
- ◯ Other ____
- ◯ Prefer not to say

Where did you obtain your primary medical qualification (PMQ)?

- ◯ UK
- ◯ European Economic Area (excluding the UK)
- ◯ Outside the UK and European Economic Area

Display This Question:

If Where did you obtain your primary medical qualification (PMQ)? = European Economic Area (excluding the UK)

Or Where did you obtain your primary medical qualification (PMQ)? = Outside the UK and European Economic Area

How many years have you practised **outside of the UK**?

- ○ Less than 5 years
- ○ 5-14 years
- ○ 15-24 years
- ○ 25-34 years
- ○ 35 years or more
- ○ Prefer not to say

Display This Question:

If Where did you obtain your primary medical qualification (PMQ)? = UK

How many years have you been practising **in the UK**?

- ○ Less than 5 years
- ○ 5-14 years
- ○ 15-24 years
- ○ 25-34 years
- ○ 35 years or more
- ○ Prefer not to say

Do you work in a primarily patient-facing role?

- Yes
- No

Display This Text:

If Do you work in a primarily patient-facing role? = No

Please be aware that if work in a non-patient facing role some questions in the survey may not feel relevant to you. If a question does not feel relevant to you/your role, please select "not applicable" as your answer option.

How old are you?

- Under 30
- 30-39
- 40-49
- 50-59
- 60 years and over
- Prefer not to say

Which of the following best describes your gender?

- Man
- Woman
- Non-binary
- My gender is not listed
- Prefer not to say

Which of the following areas do you currently practise in?

- ◯ East of England
- ◯ London
- ◯ Midlands
- ◯ North East & Yorkshire
- ◯ North West
- ◯ Northern Ireland
- ◯ Scotland
- ◯ South East
- ◯ South West
- ◯ Wales
- ◯ Prefer not to say

Questions on use of artificial intelligence systems

The following set of questions are about your use of Artificial Intelligence (AI) systems as a doctor in clinical practice.

We define these systems as **automatic or computerised systems that are trying to emulate some type of human intelligence or support some kind of intelligent decision-making or thought process**.

We are going to ask about **three areas** in which AI systems may be used to automate or augment medical or care-related tasks to understand if you have made use of them in the past twelve months. These areas are detailed in the graphic below:

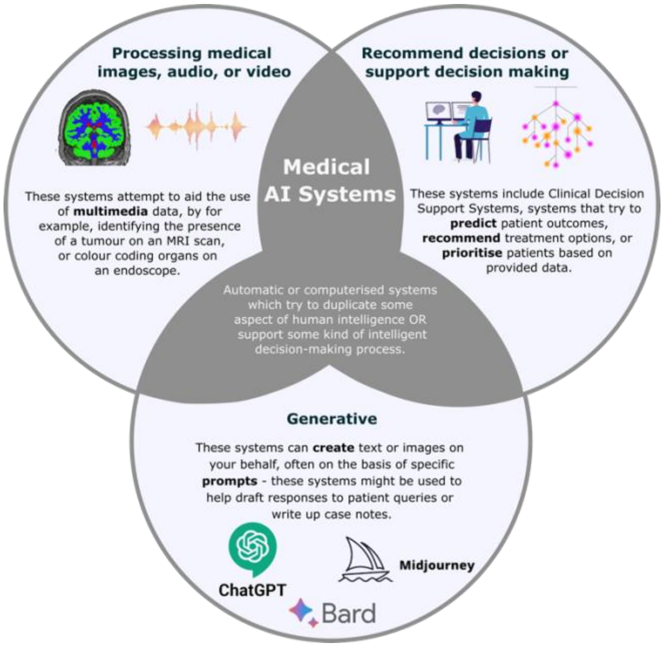

Have you used any of these, or any other, AI systems as a doctor in your practice within the last twelve months?

- ◯ Yes
- ◯ No
- ◯ I don't know

Please now select any of the systems below that you have used within the last twelve months. You may choose more than one system.

- ◯ Diagnostic and decision support system
- ◯ Generative system
- ◯ System efficiency systems
- ◯ Other system

Display This Question:

If Please now select any of the systems below that you have used within the last twelve months. You... = Other system

You have indicated that you use, or have used, a different type of system to those listed. Please provide the name(s) and/or a one sentence description of this system(s) if possible:

____

Questions for respondents who indicated they only used one of the listed AI systems

If possible, please provide the name and/or a one sentence description of the AI system that you have selected:

○ Name ____

How frequently do you use the AI system you have selected?

○ Every day

○ At least once a week

○ At least once a month

○ Less than once a month

○ I don't currently use this system in my practice

○ Other ____

Display This Question:

If How frequently do you use the AI system you have selected? = I don't currently use this system in my practice

You have selected that you do not currently use the AI system that you selected. In one sentence, please briefly describe why you no longer use the system:

____

____

____

Statements for respondents who indicated they <u>only used one</u> of the listed AI systems

The following is a set of statements regarding your use and understanding of the AI system you selected. For each statement, please select an option:

	Strongly agree	Agree	Neither agree nor disagree	Disagree	Strongly disagree	Not applicable
I feel confident using the system	○	○	○	○	○	○
The outputs of the system are clear and understandable	○	○	○	○	○	○
I have received sufficient training on the system	○	○	○	○	○	○
The system improves my clinical decision making	○	○	○	○	○	○
The system has increased my productivity	○	○	○	○	○	○
I was consulted during the deployment or integration of the AI system	○	○	○	○	○	○
I understand how to raise any concerns I have about the system	○	○	○	○	○	○
If appropriate, I could explain the outputs of the system to patients	○	○	○	○	○	○

Questions for respondents who indicated they used more than one of the listed AI systems

Please now select the **one** system that you have used **most frequently** over the past twelve months. You will then be asked a series of questions regarding your use and the impact of the system.

◯ Diagnostic and decision support system

◯ Generative system

◯ System efficiency systems

◯ Other system

If possible, please provide the name and/or a one sentence description of the AI system that you have selected as using **most frequently**:

◯ Name ____

How frequently do you use the AI system you have selected?

◯ Every day

◯ At least once a week

◯ At least once a month

◯ Less than once a month

◯ I don't currently use this system in my practice

◯ Other ____

Display This Question:

If How frequently do you use the AI system you have selected? = I don't currently use this system in my practice

You have selected that you do not currently use the AI system that you selected. In one sentence, please briefly describe why you no longer use the system:

____
____
____

Statements for respondents who indicated they used more than one of the listed AI systems

The following is a set of statements regarding your use and understanding of the AI system you have selected as using most frequently. For each statement, please select an option.

	Strongly agree	Agree	Neither agree nor disagree	Disagree	Strongly disagree	Not applicable
I feel confident using the system	○	○	○	○	○	○
The outputs of the system are clear and understandable	○	○	○	○	○	○
I have received sufficient training on the system	○	○	○	○	○	○
The system improves my clinical decision making	○	○	○	○	○	○
The system has increased my productivity	○	○	○	○	○	○
I was consulted during the deployment or integration of the AI system	○	○	○	○	○	○
I understand how to raise any concerns I have about the system	○	○	○	○	○	○
If appropriate, I could explain the outputs of the system to patients	○	○	○	○	○	○

Questions for <u>all</u> respondents on their perceptions of AI use in healthcare

The following is a set of statements regarding your **general perceptions** on the current and potential use of AI within healthcare in the UK. For each statement, please select an option.

	Strongly agree	Agree	Neither agree nor disagree	Disagree	Strongly disagree	Not applicable
AI is being deployed before it is ready in my area of practice	○	○	○	○	○	○
Opportunities for AI in healthcare are being fully explored	○	○	○	○	○	○
I understand the risks of AI in healthcare in my area of practice	○	○	○	○	○	○
Advances in AI are making me worried about my job security	○	○	○	○	○	○
Advances in AI are likely to erode my professional autonomy	○	○	○	○	○	○
Advances in AI are likely to limit training or learning opportunities	○	○	○	○	○	○

How optimistic or pessimistic are you about the integration of AI systems in healthcare/clinical practice?

- ◯ Very optimistic
- ◯ Somewhat optimistic
- ◯ Neutral
- ◯ Somewhat pessimistic
- ◯ Very pessimistic
- ◯ I don't know

The following is a set of statements regarding your perceptions on how the use of AI systems interacts with **your professional responsibilities** as a doctor. For each statement, please select an option.

	Strongly agree	Agree	Neither agree nor disagree	Disagree	Strongly disagree	Not applicable
I would feel confident to ignore the recommendations of an AI system within my area of practice	◯	◯	◯	◯	◯	◯
I understand who is responsible if a decision is made incorrectly involving an AI system	◯	◯	◯	◯	◯	◯
I have had sufficient training to understand my professional responsibilities when using AI systems	◯	◯	◯	◯	◯	◯

Scenario question to all respondents

To further explore your perceptions of AI systems, we will now present you with a hypothetical scenario and ask you to detail how you would proceed. Please review the next question and provide a response.

You are using an AI clinical decision-support system which recommends treatment options for individual patients. You disagree with the recommendation that is given by the system. How would you proceed?

Please write 'I don't know' if you do not know how you would proceed.

Please write a brief response below.

____
____
____

Is there any other information you would like to share about your experience with, or perception of, AI systems in your area of practice?

____
____
____

END OF SURVEY
