# Supplementary material for: Exploring doctors’ perspectives on generative-AI and diagnostic-decision-support systems
Source: BMJ Health Care Inform. 2025 Jul 23;32(1):e101371. doi: 10.1136/bmjhci-2024-101371 (PMC12306348; doi:10.1136/bmjhci-2024-101371)
Supplement: online supplemental file 4 [file bmjhci-32-1-s004.pdf]

Table 1, Glossary

Group	Label	Number	Question Text
perception	aiuse_perception_1	Q18	If appropriate, I could explain the outputs of the system to patients
	aiuse_perception_2	Q1	AI is being deployed before it is ready in my area of practice
	aiuse_perception_3	Q2	Opportunities for AI in healthcare are being fully explored
	aiuse_perception_4	Q3	I understand the risks of AI in healthcare in my area of practice
	aiuse_perception_5	Q4	Advances in AI are making me worried about my job security
	aiuse_perception_6	Q5	Advances in AI are likely to erode my professional autonomy
	aiuse_optimism	Q6	Advances in AI are likely to limit training or learning opportunities
Responsibility	aiuse_impact_1	Q7	How optimistic or pessimistic are you about the integration of AI systems in healthcare/clinical practice
	aiuse_impact_2	Q8	I would feel confident to ignore the recommendations of an AI system within my area of practice
	aiuse_impact_3	Q9	I understand who is responsible if a decision is made incorrectly involving an AI system
Experience	ai_use_confidence	Q10	I have had sufficient training to understand my professional responsibilities when using AI systems
	ai_use_clear_outputs	Q11	I feel confident using the system
	ai_use_training	Q12	The outputs of the system are clear and understandable
	ai_use_decision_making	Q13	I have received sufficient training on the system
	ai_use_productivity	Q14	The system improves my clinical decision making
	ai_use_consulted	Q15	The system has increased my productivity
	ai_use_concerns	Q16	I was consulted during the deployment or integration of the AI system
	ai_use_explain	Q17	I understand how to raise any concerns I have about the system

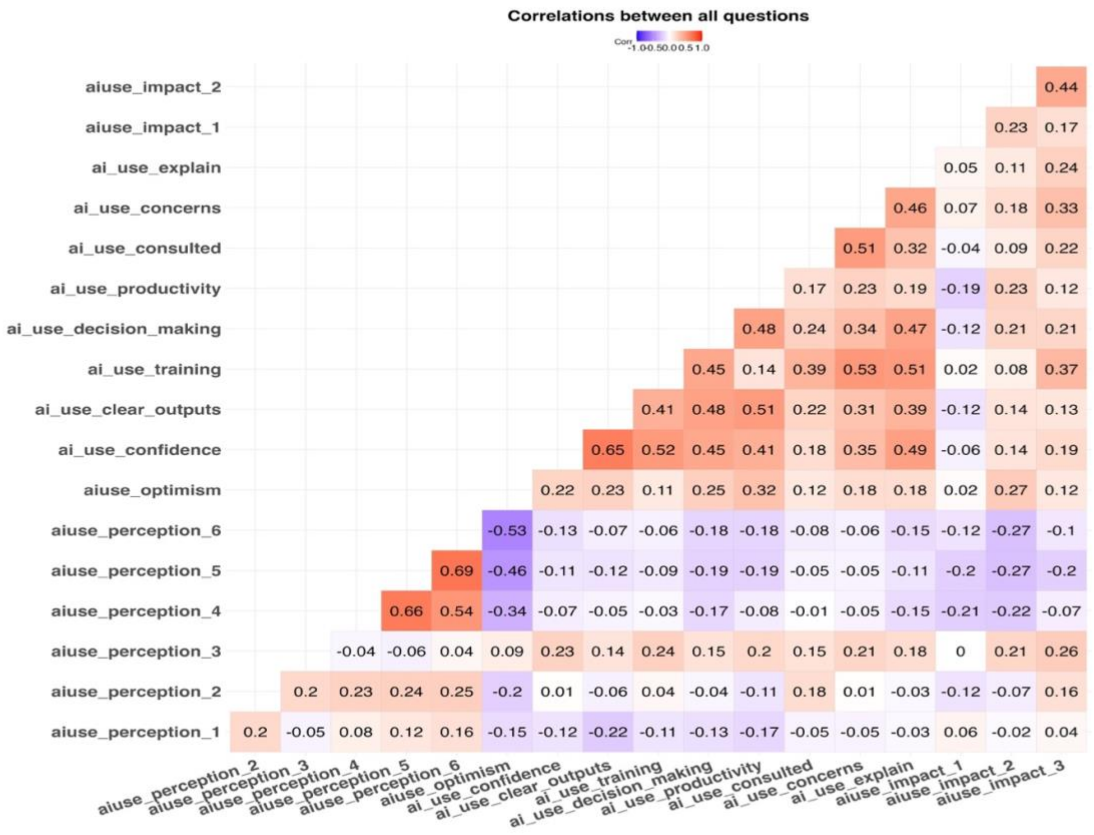

Table 2, Questions grouped by demographics
(numbers are % of respondents in each sub- group).

**Group**	**Sub-group**	**Choice**	Q1	Q2	Q3	Q4	Q5	Q6	Q7	Q8	Q9	Q10	Q11	Q12	Q13	Q14	Q15	Q16	Q17	Q18
All	All	Agree	13	14	44	15	26	28	24	54	30	12	72	81	38	42	53	18	35	53
		Disagree	38	54	32	67	50	51	52	20	51	70	5	7	29	17	16	37	35	12
		Neutral	49	32	24	19	24	21	24	26	19	18	23	12	33	41	31	45	29	35
Registration Status	GP	Agree	10	14	39	17	33	23	55	66	31	8	77	86	47	51	54	25	38	57
		Disagree	37	49	38	67	44	55	23	16	52	77	8	7	29	19	20	38	34	8
		Neutral	53	37	23	16	23	22	22	18	17	15	15	7	24	30	26	37	28	35
	LED and SAS	Agree	16	16	48	16	28	37	47	42	35	15	75	79	32	38	58	14	37	49
		Disagree	32	52	25	57	43	44	22	29	46	62	4	4	30	11	3	33	37	16
		Neutral	52	32	27	27	29	19	31	29	19	23	21	17	38	51	39	53	26	35
	Specialist	Agree	15	12	40	10	20	23	56	60	28	14	72	86	48	45	49	25	40	59
		Disagree	44	59	32	78	59	55	25	17	51	72	6	9	24	15	22	35	28	11
		Neutral	41	29	28	12	21	22	19	23	21	14	22	5	28	40	29	40	32	30
	Trainee	Agree	8	14	48	17	26	25	49	50	29	8	62	71	18	30	54	3	22	44
		Disagree	39	54	33	64	51	51	27	18	54	70	2	6	38	28	19	46	47	13
		Neutral	53	32	19	19	23	24	24	32	17	22	36	23	44	42	27	51	31	43
Age	40-49	Agree	14	14	40	15	24	27	57	52	26	12	62	82	39	39	50	19	32	55
		Disagree	38	53	31	70	53	54	22	20	53	73	10	9	30	17	22	31	34	14
		Neutral	48	33	29	15	23	19	21	28	21	15	28	9	31	44	28	50	34	31
	50+	Agree	16	12	40	11	27	23	53	58	31	15	79	78	59	50	50	29	50	67
		Disagree	37	47	32	71	51	52	24	18	49	68	5	11	18	18	23	34	22	6
		Neutral	47	41	28	18	22	25	23	24	20	17	16	11	23	32	27	37	28	27
	Under 40	Agree	11	15	48	16	27	30	49	53	32	10	74	82	29	40	56	13	31	47
		Disagree	38	58	31	63	47	49	25	21	50	69	3	4	33	17	10	41	41	13
		Neutral	51	27	21	21	26	21	26	26	18	21	23	14	38	43	34	46	28	40
Speciality	Anaesthetics and Intensive Care Medicine	Agree	15	6	27	13	25	21	41	54	32	8	60	77	39	43	40	15	31	52
		Disagree	38	56	46	73	61	60	27	21	51	79	40	4	41	28	31	41	23	16
		Neutral	47	38	27	14	14	19	32	25	17	13	0	19	20	29	29	44	46	32
	Emergency Medicine	Agree	18	13	51	18	34	46	40	38	26	12	80	100	57	39	61	15	39	55
		Disagree	38	51	24	55	42	27	32	33	53	70	5	0	9	27	11	41	39	30
		Neutral	44	36	25	27	24	27	28	29	21	18	15	0	34	34	28	44	22	15
	General Practice	Agree	9	15	43	17	28	22	56	64	33	9	72	84	46	51	58	26	37	57
		Disagree	37	49	38	67	49	58	22	18	48	73	8	7	28	16	17	35	36	6
		Neutral	54	36	19	16	23	20	22	18	19	18	20	9	26	33	25	39	27	37
	Medicine	Agree	10	13	42	14	25	29	45	53	27	15	72	75	33	43	46	15	32	44
		Disagree	39	53	28	65	47	47	26	17	55	62	4	7	31	14	15	34	42	10
		Neutral	51	34	30	21	28	24	29	30	18	23	24	18	36	43	39	51	26	46
	Paediatrics	Agree	15	13	36	14	30	30	56	40	31	13	54	76	38	27	71	51	18	30
		Disagree	47	60	30	72	45	50	24	33	49	71	30	14	47	17	17	49	50	46

		Neutral	38	27	34	14	25	20	20	27	20	16	16	10	15	56	12	0	32	24
	Psychiatry	Agree	4	16	50	11	23	25	53	59	22	7	50	76	21	27	44	21	21	44
		Disagree	36	50	35	75	62	54	30	17	64	73	8	24	39	73	24	39	35	16
		Neutral	60	34	15	14	15	21	17	24	14	20	42	0	40	0	32	40	44	40
	Radiology	Agree	10	23	50	11	19	22	82	58	26	11	76	85	41	23	52	23	46	76
		Disagree	54	58	24	59	64	60	10	16	51	60	4	7	25	25	20	55	48	16
		Neutral	36	19	26	30	17	18	8	26	23	29	20	8	34	52	28	22	6	8
	Surgery	Agree	22	16	47	11	24	32	57	50	31	12	71	79	25	43	48	16	34	57
		Disagree	37	63	32	74	50	51	21	21	51	79	3	7	28	12	13	40	33	4
		Neutral	41	21	21	15	26	17	22	29	18	9	26	14	47	45	39	44	33	39
PMQ	EEA	Agree	17	12	55	15	22	22	56	55	37	25	80	93	35	44	65	16	41	67
		Disagree	33	53	23	62	55	51	20	18	42	61	6	4	32	23	7	51	30	17
		Neutral	50	35	22	23	23	27	24	27	21	14	14	3	33	33	28	33	29	16
	IMG	Agree	16	18	49	16	24	34	54	42	41	16	73	78	33	50	70	13	33	47
		Disagree	31	53	19	59	46	47	16	26	33	57	3	4	32	11	4	32	38	12
		Neutral	53	29	32	25	30	19	30	32	26	27	24	18	35	39	26	55	29	41
	UK	Agree	10	11	39	14	28	25	50	60	23	8	70	82	42	35	39	22	37	56
		Disagree	43	55	40	72	51	53	29	18	62	78	7	9	26	21	26	39	34	11
		Neutral	47	34	21	14	21	22	21	22	15	14	23	9	32	44	35	39	29	33
Gender	Female	Agree	12	14	38	15	27	28	43	47	25	10	67	82	39	41	44	17	34	48
		Disagree	33	51	35	64	44	48	26	23	56	72	5	4	28	14	19	42	43	12
		Neutral	55	35	27	21	29	24	31	30	19	18	28	14	33	45	37	41	23	40
	Male	Agree	13	14	50	15	25	28	61	61	35	13	76	81	37	42	61	18	36	57
		Disagree	43	57	28	69	55	54	22	18	46	68	6	8	29	20	13	34	30	12
		Neutral	44	29	22	16	20	18	17	21	19	19	18	11	34	38	26	48	34	31
